# The Association between Vitamin D and Vascular Stiffness in Adolescents with and without Type 1 Diabetes

**DOI:** 10.1371/journal.pone.0077272

**Published:** 2013-10-29

**Authors:** Rachel Lieberman, R. Paul Wadwa, Nhung Nguyen, Franziska K. Bishop, Christina Reinick, Janet K. Snell-Bergeon, David M. Maahs

**Affiliations:** 1 Saint Louis University School of Medicine, St. Louis, Missouri, United States of America; 2 Barbara Davis Center for Childhood Diabetes, University of Colorado Anschutz Medical Campus, Aurora, Colorado, United States of America; 3 Children's Hospital Colorado, Aurora, Colorado, United States of America; 4 Department of Epidemiology, Colorado School of Public Health, University of Colorado Denver, Aurora, Colorado, United States of America; Washington Hospital Center, United States of America

## Abstract

**Objective:**

Vitamin D deficiency is common and associated with increased cardiovascular disease (CVD) risk. Pulse wave velocity (PWV) is a marker of vascular stiffness associated with CVD. We hypothesized that Vitamin D (25 (OH) D) levels would be inversely associated with PWV in youth with and without type 1 diabetes (T1D).

**Study Design:**

Comparisons were made between adolescents with T1D (n = 211; age = 17.5±2.3 years; diabetes duration = 10.9±3.2 years; A1c = 9.1±1.7%) and non-DM controls (n = 67; age = 16.9±1.9 years). PWV was measured in the carotid-femoral segment (Sphygmocor Vx, AtCor Medical, Lisle, IL).

**Results:**

Vitamin D levels were similar in adolescents with T1D and controls (27.7±0.7 v. 26.0±1.3 ng/ml; p = 0.26). Vitamin D was significantly inversely associated with PWV after adjusting for age, sex, quarter of the year, and race-ethnicity in adolescents with T1D (beta  = −0.01±0.004, p = 0.02) but not in the non-DM adolescents (beta  = −0.008±0.008, p = 0.32). Vitamin D remained significantly associated with PWV after additionally adjusting for hs-CRP in adolescents with T1D (−0.01±0.004, p = 0.01). After adjusting for BMI z-score, lipids, or blood pressure, the relationship of Vitamin D with PWV was not significant.

**Conclusions:**

Vitamin D levels were inversely associated with PWV in adolescents with T1D, but not independently of BMI, lipids, or blood pressure. Our data contrast with other reports and suggest further research is indicated to determine if Vitamin D supplementation would be beneficial to lower CVD risk in adolescents with T1D with vitamin D insufficiency or deficiency.

## Introduction

Vitamin D insufficiency and deficiency are common in the United States, particularly in the adolescent population, in which half of adolescents have been shown to be insufficient in vitamin D [1]. This lack of sufficient vitamin D is associated with increased cardiovascular disease (CVD) risk and has been linked to major cardiometabolic risk factors, such as obesity, high blood pressure, and fasting hyperglycemia in adolescents [2], raising concern about the future health risks associated with this widespread hypovitaminosis D.

Observational studies have suggested that Vitamin D-deficient individuals are at an increased risk for the development of autoimmune disease, including Type 1 diabetes (T1D) [3], [4]. Further, vitamin D insufficiency is prevalent in children and adolescents with T1D [5], [6]. Most studies comparing vitamin D levels between pediatric T1D patients and non-diabetic (non-DM) controls have shown no significant difference between the two groups [7], [8]. One study showed lower levels of vitamin D in youth with new-onset T1D than in non-diabetic youth [9].

Pulse wave velocity (PWV) is a marker of vascular stiffness associated with CVD, and vitamin D deficiency is inversely associated with PWV in non-diabetic adults [10], [11]. Adults with T1D have increased PWV compared to age-matched, non-DM controls [12]. The same has shown to be true in adolescents with T1D [13]. However, no study has investigated the association between serum Vitamin D levels and arterial stiffness in the T1D adolescent population. We hypothesized that serum Vitamin D (25 (OH) D) levels would be inversely associated with PWV in a cohort of adolescents with and without T1D.

## Methods

### Ethics Statement

The study was approved by the Colorado Multiple Institution Review Board. Written informed consent was obtained from all subjects over 18 years old or parent or legal guardian and written informed assent was obtained from all subjects less than 18 years old before participation in the study. The study was conducted in accordance with the Declaration of Helsinki guidelines.

### Study population

The Determinants of Macrovascular Disease in Adolescents with T1D study was initiated to investigate atherosclerotic disease risk in youth with and without T1D. Subjects were 12–19 years of age. Study participants with T1D were diagnosed by islet cell antibody or by provider clinical diagnosis, had diabetes duration >5 years at entry into the study, and received clinical care at the Barbara Davis Center for Childhood Diabetes (BDC). Non-DM control subjects were recruited from friends of the study subjects as well as from campus and community advertisements. No siblings of patients with T1D were included. Subjects were excluded for diabetes of any other type and for any history of abnormal cardiac anatomy or arrhythmia that would preclude the subject from vascular function measurements. The study was approved by the Colorado Multiple Institution Review Board and informed consent and assent (for subjects <18 years) was obtained from all subjects. All data were from the second study visit (2010–2012) in this longitudinal study.

### Visit Summary

All subjects fasted overnight (>8 hours) and were asked to refrain from caffeine intake and smoking within 8 hours prior to study visit (due to potential effect on vascular measures). Tanner Stage for all BDC patients was assessed by a pediatric endocrinologist. Tanner stage for non-DM subjects was assessed with a physical exam by a pediatric endocrinologist with the option of self-assessment if the subject refused a physical exam. After subjects had been laying supine for a minimum of 5 minutes, blood pressure measurements were obtained using a Dynapulse 5200A (Pulse Metric, Inc., San Diego, CA) and 3 measurements were averaged. Height was measured to the nearest 0.1 cm with shoes removed using a wall-mounted stadiometer, and weight was measured to the nearest 0.1 kg using a Detecto scale. BMI was calculated and in subjects <20 years of age BMI z-score was calculated using the 2000 Centers for Disease Control and Prevention growth chart standards (n = 42 without BMI z-score). Waist circumference was measured at the navel on bare skin using the Figure Finder Tape Measure by Novel Products, Inc (Rockton, IL), which provides consistent and repeatable 4 oz. of tension and accuracy to 3/32 inch. PWV was measured in the carotid-femoral segment, and was obtained using arterial tonometry with the Sphygmocor Vx (AtCor Medical, Lisle, IL). The distance from the lowest portion of the sternal notch to the artery recorded (femoral) was measured to the nearest 0.1 cm twice, averaged and entered into the SphygmoCor software. ECG leads were applied and arterial waveforms were recorded with a high-fidelity micromanometer from the carotid pulse. A second arterial waveform was recorded from the femoral artery. The waveforms were gated by the R-wave on the simultaneously recorded ECG. PWV is determined by calculation of the difference in the carotid-to-femoral (or radial or dorsalis pedis) path length divided by the difference in R-wave-to-waveform foot times. The average of 10 successive measurements was used in the analyses to cover a complete respiratory cycle. The procedure was repeated 3 times per location per subject and mean PWV for each location was used for analysis.

### Laboratory Assays

HbA1c was measured on the DCA Advantage by Siemens at the Children's Hospital Colorado main clinical lab. Total cholesterol (TC), HDL-cholesterol (HDL-c), and triglycerides (TG) were performed in the Clinical Translational Research Core (CTRC) lab using an Olympus AU400e Chemistry. LDL-cholesterol (LDL-c) was calculated using the Friedewald formula. hs-CRP was measured at the Children's Hospital Colorado CTRC core lab utilizing a multiplex assay platform Siemens (formally Dade Behring) BNII Nephelometer. Vitamin D was measured at the Children's Hospital Colorado CTRC lab utilizing a Vitamin D 25 OH assay in samples from the second study visit. The serum level of 25 (OH) D was then categorized using the Institute of Medicine guidelines, which define Vitamin D deficiency as <20 ng/ml; insufficiency as 20–29 ng/ml; and sufficiency as ≥30 ng/ml [14].

### Statistical Methods

Clinical characteristics were compared between T1D and non-DM adolescents using t-tests for continuous variables and chi-square tests for categorical variables. Vitamin D levels were compared between adolescents with T1D and non-T1D controls in each of the three categories: deficient, insufficient, and sufficient using a categorical Chi-square test. Vitamin D covariates were compared between adolescents with T1D and non-DM controls using Pearson and/or Spearman correlation testing. Correlation coefficients and linear regression analyses were also performed with p<0.05 considered as statistically significant.

## Results

Clinical characteristics were compared between adolescents with T1D and non-DM controls (Table 1). The adolescents with T1D were slightly older than the non-DM adolescents (17.6±2.3 years vs. 16.9±1.9 years; p = 0.04), and had significantly worse lipid profiles. Both triglycerides (geometric mean 101 mg/dl (range 37–1532) vs. 74 mg/dl (28–246); p = 0.01) and LDL-C (93±30 mg/dl vs. 83±22 mg/dl; p = 0.003) were significantly higher in the adolescents with T1D. Blood pressure in adolescents with T1D was also significantly higher compared to the non-DM controls (SBP: 116±10 mmHg vs. 112±8 mmHg; p = 0.0007 and DBP: 71±7 mmHg vs. 67±6 mmHg; p<0.0001). PWV was significantly higher in adolescents with T1D vs. non-DM controls (5.8±0.7 vs. 5.5±0.7; p = 0.007).

**Table 1:** Clinical characteristics of study populations.

	T1D (N = 211)	Non-DM (N = 67)	P-value
**Age,** y	17.6±2.3	16.9±1.9	0.04
**Sex,** % male	51	52	0.88
**Race-Ethnicity,** % NHW	86	73	0.014
**T1D Duration,** y	10.9±3.2	N/A	<0.0001
**Treatment Type,** %			
Injection	39	N/A	N/A
Pump	61	N/A	N/A
**Tanner Stage,** n, %			0.36
I	0, 0	0, 0	
II	2, 1	1, 2	
III	14, 8	1, 2	
IV	40, 22	16, 26	
V	124, 69	43, 71	
**HbA1c,** %	9.1±1.7	5.2±0.2	<0.0001
**BMI,** kg/m^2^	23.8±3.6	22.8±5.1	0.13
**BMI z-score**	0.6±0.8	0.2±1.1	0.04
**Total cholesterol,** mg/dl	165±39	147±30	<0.0001
** **Triglycerides,** mg/dl	101 (37–1532)	74 (28–246)	0.01
**HDL-c,** mg/dl	51±11	48±12	0.02
**LDL-c,** mg/dl	93±30	83±22	0.003
**SBP,** mmHg	116±10	112±8	0.0007
**DBP,** mmHg	71±7	67±6	<0.0001
** **hs-CRP,** mg/dl	0.92 (0.06–31.9)	0.6 (0.03–20.1)	0.0523
**Vitamin D,** ng/ml (unadjusted)	27.6±11.1	25.6±10.9	0.2
**Vitamin D,** ng/ml (*adjusted for quarter of the year)	27.7±0.7	26.0±1.3	0.26
**PWV,** m/sec (unadjusted)	5.8±0.7	5.5±0.7	0.007

* LS means ± standard error.

** Geometric mean and range.

*** N = 211 for T1D and N = 67 for Non-DM except for NHW (T1D = 209, Control = 67); Tanner Stage (T1D = 180, Control = 61); HbA1C (T1D = 210, Control = 65); BMI z-score (T1D = 174, Control = 62, n = 42 older than 20 years of age); Total cholesterol, Triglycerides, HDL-c (T1D = 209, Control = 66); LDL-c (T1D = 208, Control = 66); hsCRP (T1D = 208, Control = 65); Vitamin D (T1D = 207, Control = 66); PWV (T1D = 202, Control = 67).

**Figure 1:**
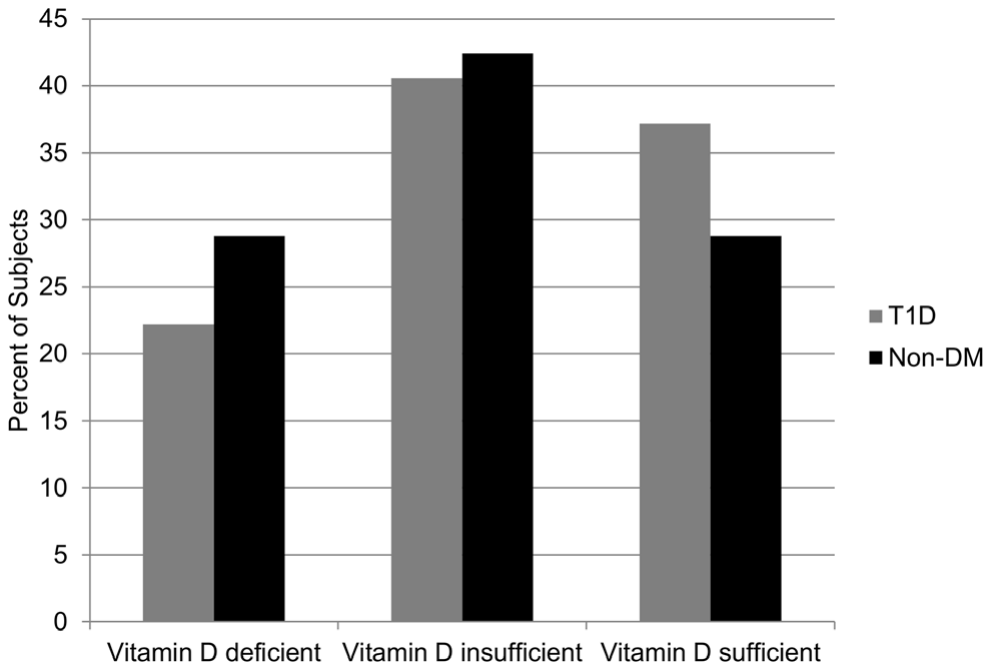
Vitamin D levels categorized and compared between adolescents with T1D and controls. Categorization of Vitamin D based on the Institute of Medicine guidelines (Mantel-Haenszel p-value = 0.16).

Vitamin D levels did not differ in adolescents with T1D vs. non-DM controls (27.6±11.1 ng/ml vs. 25.6±10.9 ng/ml; p = 0.2), and remained similar after adjusting for quarter of the year (Table 1). When Vitamin D was categorized into deficiency, insufficiency, and sufficiency between adolescents with T1D and the non-DM controls, the distribution was not significantly different (p = 0.16) (Figure 1).

In univariate analysis, PWV was significantly correlated to vitamin D levels in adolescents with T1D (r = −0.19, p = 0.007), but not in non-DM adolescents (r = −0.06, p = 0.62) (Table 2). In multiple linear regression analyses (Table 3), vitamin D was significantly associated with PWV after adjusting for age, sex, quarter of the year, and race-ethnicity in adolescents with T1D (−0.01±0.004, p = 0.02), but not in the non-DM controls (−0.008±0.008, p = 0.32). Vitamin D also remained significantly associated with PWV after additionally adjusting for hs-CRP in adolescents with T1D (−0.01±0.004, p = 0.01). After adjusting for BMI z-score, lipids (TC, LDL-C, HDL-C, and TG), or blood pressure, the relationship of vitamin D with PWV was not significant. Analyzed categorically, vitamin D was not significantly associated with PWV.

**Table 2:** Correlations of Vitamin D to potentially confounding variables.

	T1D		Non-DM	
	r	P-value	r	P-value
**Age,** y	−0.02	0.77	0.18	0.14
**Sex,** % male	−0.04	0.57	0.2	0.1
**T1D duration,** y	−0.04	0.6	N/A	N/A
**HbA1c,** %	−0.28	<0.0001	−0.12	0.35
**BMI,** kg/m^2^	−0.16	0.02	−0.34	0.005
**BMI z-score**	−0.12	0.13	−0.3	0.02
**Total cholesterol,** mg/dl	−0.32	<.0001	0.03	0.79
**Triglycerides,** mg/dl	−0.27	0.0001	−0.08	0.54
**HDL-c**, mg/dl	−0.09	0.2	0.18	0.15
**LDL-c,** mg/dl	−0.24	0.0004	−0.04	0.73
**SBP,** mmHg	−0.18	0.009	−0.09	0.48
**DBP,** mmHg	−0.23	0.008	−0.13	0.32
**hsCRP,** mg/dl	0.11	0.13	−0.08	0.52
**PWV,** m/sec	−0.19	0.0071	−0.06	0.62

**Table 3:** Linear Regression Models for Vitamin D as a predictor of PWV.

	T1D (N = 211)		Non-DM (N = 67)	
	β± SE	P-value	β± SE	P-value
**Model 1**: Vitamin D adjusted for age, sex, quarter of the year, and race-ethnicity	−0.01±0.004	0.02	−0.008±0.008	0.32
**Model 2:** Model 1± hs-CRP	−0.01±0.004	0.01	−0.007±0.008	0.39
**Model 3:** Model 1± BMI z-score	−0.005±0.005	0.31	−0.003±0.008	0.68
**Model 4:** Model 1± TC, LDL-C, HDL-C, and TG	−0.007±0.004	0.14	−0.008±0.008	0.32
**Model 5:** Model 1± SBP and DBP	−0.005±0.004	0.17	−0.006±0.007	0.44

## Conclusions

These data show that PWV was significantly higher in adolescents with T1D compared to similarly aged non-DM controls, and was inversely associated with vitamin D levels in adolescents with T1D. After adjustment for significant confounding variables (age, sex, quarter of the year, race-ethnicity, and hs-CRP), low serum vitamin D levels remain significantly associated with an increased PWV in adolescents with T1D. However, the association between vitamin D and PWV did not exist after adjustment for other CVD risk factors. This difference in association of vitamin D to PWV in adolescents with T1D and non-DM controls, may, however, be due to the lack of power in the non-DM control group. One could also speculate that the association may differ in adolescents with T1D due to unknown mechanisms.

Vitamin D insufficiency is prevalent in children and adolescents with T1D [5], [6], but we found no significant difference in Vitamin D levels between adolescents with T1D and non-DM controls. Even in Colorado, which has at least 300 days of sunshine a year, Vitamin D deficiency and insufficiency is still very prevalent (Figure 1) [15].

Vitamin D has been shown to be inversely associated with HbA1c in both the adult and pediatric population; as well as with BMI in both adults [16]–[18] and youth [19]; triglycerides [17], [20], [21] and systolic blood pressure [20], [22], [23] in the adult population; and hs-CRP [24] in the pediatric population. High vitamin D concentrations are associated with a more favorable serum lipid profile [17], [20], [21], [25], [26].

Vitamin D deficiency was associated with the presence of coronary artery calcification (CAC) in adults with T1D [27]. This is significant because it extends the association between vitamin D deficiency and CVD risk factors in patients with T1D to markers of atherosclerosis. In patients with type 2 diabetes (T2D), it has been shown that vitamin D prevents foam cell formation by suppressing cholesterol uptake, and reverses cholesterol deposition in macrophages [28]. These findings point to the possibility of using vitamin D as a therapy for atherosclerosis. This is significant, as coronary artery disease is one of the major causes of mortality in the United States and the leading cause of mortality in adults with diabetes. One 15-year longitudinal study found that very low levels of plasma vitamin D were an independent predictor of all-cause mortality in their T2D subject population [29]. This study also demonstrated that low levels of vitamin D were predictive of cardiovascular mortality [29]. However, this topic requires further study as the DCCT-EDIC study found no association between lower plasma vitamin D metabolites and an increased risk of CVD [30] as measured by CAC and carotid intima-media thickness (cIMT). Clinical trials using vitamin supplementation to reduce cardiovascular mortality have not achieved positive outcomes previously [31], despite positive associations in epidemiologic studies [32], [33]. Negative reports such as ours are important to consider prior to embarking on a clinical trial.

Our data have limitations to consider. First, the data are cross-sectional, as serum vitamin D levels were only obtained at the two-year follow-up visit of a longitudinal study [34]. Second, our cohort is predominantly non-Hispanic white (86% T1D; 73% non-DM controls); however, this is representative of the T1D population in Colorado. We used a surrogate marker of CVD risk, PWV, although use of surrogate measures of atherosclerosis is necessary in adolescents. PWV has been used extensively and is associated with CVD outcomes [35].

In conclusion, vitamin D deficiency and insufficiency is common in adolescents with and without T1D, but these data suggest that vitamin D deficiency is no more common in adolescents with T1D compared to non-DM controls. These results also suggest that vitamin D is inversely associated with increased vascular stiffness, a marker of CVD, in youth with T1D, but not independently of known CVD risk factors. Furthermore, vitamin D may have a role as a marker of increased CVD risk in addition to traditional CVD markers, especially in adolescents with T1D who have a higher lifetime risk of CVD. Further research is needed to determine if Vitamin D supplementation would be beneficial to lower CVD risk in adolescents with T1D.
